# Supplementary figures and images for: Different effects of plasmids harboring blaOXA-232 between major and minor clones in Klebsiella pneumoniae
Source: Microbiol Spectr. 2025 Jun 12;13(8):e02126-24. doi: 10.1128/spectrum.02126-24 (PMC12323343; doi:10.1128/spectrum.02126-24)

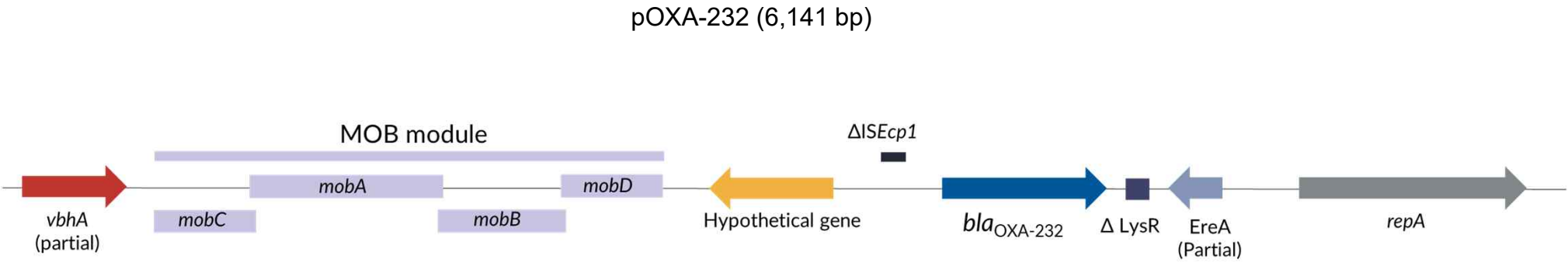

**Supplementary Figure S1.** A linear map of pOXA-232 indicating the plasmid genes used in this study.

Supplement: Fig. S1 — A linear map of pOXA-232 indicating the plasmid genes used in this study. [file spectrum.02126-24-s0001.pdf]
