# Supplementary material for: Different effects of plasmids harboring blaOXA-232 between major and minor clones in Klebsiella pneumoniae
Source: Microbiol Spectr. 2025 Jun 12;13(8):e02126-24. doi: 10.1128/spectrum.02126-24 (PMC12323343; doi:10.1128/spectrum.02126-24)
Supplement: Supplemental tables — Tables S1 and S2. [file spectrum.02126-24-s0002.pdf]

Supplementary Table S1. Carbapenem susceptibility profile for wild-type and intact plasmid transconjugants.

	Genotype	Isolate number	MIC (mg/L)			
			Imipenem		Meropenem	
			WT	T-pOXA-232	WT	T-pOXA232
**Major clones**	ST11	KCS01	0.25	4	0.125	4
		KCS02	0.25	4	0.125	4
		KCS03	0.125	4–8	0.125	4
		KCS04	0.25	8	0.25	4
		KCS05	0.25	4	0.125	4
		KCS06	0.25	4–8	0.125	8
		KCS07	0.25	4	0.125	4
		KCS08 *	0.5	64	0.5	64
		KCS09	1	4	0.25	8
		KCS10 *	0.125	4	0.06	4
		KCS11	0.125	4	0.25	4
		KCS12	0.25	2	0.06	4
		KCS13	1	2–4	0.25	4
		KCS14	2	4	0.25	4
		KCS15	1	2–4	0.25	8
		KCS16	0.25	4–8	0.25	4
		KCS17	1	2	0.25	8
		KCS18	0.25	4	0.25	8
		KCS19 *	0.25	8–16	0.125	16
		KCS21	0.06	8–16	0.03	16
		KCS23	0.5	4	0.125	4
	ST15	KCS24	0.25	4	0.125	4
		KCS25	0.125	2–4	0.06	4
		KCS26 *	0.5	2	0.5	2
		KCS27 *	0.5	4	0.5	4
	ST307	KCS29 *	0.25	4	0.06	4
		KCS30 *	0.125	2–4	0.125	4
		KCS31	0.25	2	0.06	4
		KCS32	0.25	4	0.06	4
**Minor clones**	ST23	KCS28	0.06	4	0.06	4
	ST27	KCS33 *	0.06	2	0.06	2
	ST105	KCS34	0.5	4	0.06	4
	ST165	KCS35	0.5	4	0.06	4
	ST298	KCS36 *	1	4	0.06	4
	ST355	KCS37	0.25	4	0.06	4
	ST356	KCS38	0.125	4–8	0.06	4
	ST358	KCS39	0.5	4–8	0.06	4
	ST365	KCS40	0.25	4	0.06	2
	ST469	KCS41 *	0.5	4	0.06	4

*Isolates used for further studies, including competition and human serum assays.

Supplementary Table S2. Primers used in this study.

Primers	Sequences (5′ →3′)	T_m (°C)	References
For carbapenemase gene detection			
KPCtype-F	TGTTGCTGAAGGAGTTGGGC	56	[17]
KPCtype-R	ACGACGGCATAGTCATTTGC	56	
NDM-F	GGTTTGGCGATCTGGTTTTC	55	[17]
NDM-R	CGGAATGGCTCATCACGATC	57	
OXA-48-like-F	TGAGCACTTCTTTTGTGATGGCT	61	[17]
OXA-48-like-R	AACGGGCGAACCAAGCATTTT	59	
CTX-M-F	CRATGTGCAGYACCAGTAA	51	[17]
CTX-M-R	CGCRATATCRTTGGTGGTG	54	
OXA-232-FW	GGCTGTGTTTTTGGTGGCAT	58	This study
OXA-232-RV	CCCAAAGCGCCTCTGTAGAA	59	
For qRT-PCT			
qRT_*fimH*_FW	GACCAACAACTACAATAGCGAC	61	This study
qRT_*fimH*_RV	ATTGGTGAAGATCGCGTTGG	58	This study
